# HIV-1 transmitted drug resistance in newly diagnosed individuals in Italy over the period 2015–21

**DOI:** 10.1093/jac/dkae189

**Published:** 2024-07-19

**Authors:** Lavinia Fabeni, Daniele Armenia, Isabella Abbate, Roberta Gagliardini, Valentina Mazzotta, Ada Bertoli, William Gennari, Federica Forbici, Giulia Berno, Lorenzo Piermatteo, Vanni Borghi, Carmela Pinnetti, Alessandra Vergori, Annalisa Mondi, Giustino Parruti, Fiorella Di Sora, Marco Iannetta, Miriam Lichtner, Alessandra Latini, Cristina Mussini, Loredana Sarmati, Carlo Federico Perno, Enrico Girardi, Andrea Antinori, Francesca Ceccherini-Silberstein, Fabrizio Maggi, Maria Mercedes Santoro, The Italian HIV Drug Resistance Group, F Ceccherini-Silberstein, M C Bellocchi, L Carioti, M M Santoro, M Andreoni, M Iannetta, A Bertoli, L Sarmati, V Malagnino, E Teti, D Armenia, A Antinori, F Baldini, R Bellagamba, G Berno, M Camici, S Cicalini, F De Zottis, R Esvan, L Fabeni, F Forbici, M Fusto, R Gagliardini, S Gebremeskel, F Gili, E Girardi, E Grilli, S Grisetti, I Mastrorosa, V Mazzotta, A Mondi, N Orchi, S Ottou, C Pinnetti, S Pittalis, D Pizzi, M Plazzi, A Vergori, A R Buonomini, M Giuliani, A Latini, A Pacifici, C F Perno, V Belvisi, C Del Borgo, A Carraro, M Lichtner, R Marocco, V Borghi, C Mussini, W Gennari

**Affiliations:** Laboratory of Virology, National Institute for Infectious Diseases, Lazzaro Spallanzani IRCCS, Rome, Italy; Departmental Faculty, UniCamillus, Saint Camillus International University of Health Sciences, Rome, Italy; Laboratory of Virology, National Institute for Infectious Diseases, Lazzaro Spallanzani IRCCS, Rome, Italy; Clinical and Research Infectious Diseases Department, National Institute for Infectious Diseases, Lazzaro Spallanzani IRCCS, Rome, Italy; Clinical and Research Infectious Diseases Department, National Institute for Infectious Diseases, Lazzaro Spallanzani IRCCS, Rome, Italy; Laboratory of Virology, Department of Laboratory Medicine, University Hospital Tor Vergata, Rome, Italy; Molecular Microbiology and Virology Unit, Department of Laboratory Medicine and Pathological Anatomy, Policlinic of Modena, University of Modena and Reggio Emilia, Modena, Italy; Laboratory of Virology, National Institute for Infectious Diseases, Lazzaro Spallanzani IRCCS, Rome, Italy; Laboratory of Virology, National Institute for Infectious Diseases, Lazzaro Spallanzani IRCCS, Rome, Italy; Department of Biology, University of Rome Tor Vergata, Rome, Italy; Department of Infectious Diseases, Azienda Ospedaliero-Universitaria, Policlinico of Modena, Modena, Italy; Clinical and Research Infectious Diseases Department, National Institute for Infectious Diseases, Lazzaro Spallanzani IRCCS, Rome, Italy; Clinical and Research Infectious Diseases Department, National Institute for Infectious Diseases, Lazzaro Spallanzani IRCCS, Rome, Italy; Clinical and Research Infectious Diseases Department, National Institute for Infectious Diseases, Lazzaro Spallanzani IRCCS, Rome, Italy; Infectious Diseases Unit, Pescara General Hospital, Pescara, Italy; Unit of Clinical Immunology, San Giovanni Addolorata Hospital, Rome, Italy; Department of Infectious Diseases, University Hospital Tor Vergata, Rome, Italy; Infectious Diseases Unit, Santa Maria Goretti Hospital, Sapienza University of Rome, Polo Pontino, Latina, Italy; Sant'Andrea Hospital, Clinical Infectious Diseases, Rome, Italy; Sexually Transmitted Infection/Human Immunodeficiency Virus Unit, San Gallicano Dermatological Institute IRCCS, Rome, Italy; Department of Infectious Diseases, Azienda Ospedaliero-Universitaria, Policlinico of Modena, Modena, Italy; Department of Infectious Diseases, University Hospital Tor Vergata, Rome, Italy; Microbiology and Diagnostic Immunology Unit, Department of Diagnostic and Laboratory Medicine, Bambino Gesú Children's Hospital, IRCCS, Rome, Italy; Scientific Direction, National Institute for Infectious Diseases, Lazzaro Spallanzani IRCCS, Rome, Italy; Clinical and Research Infectious Diseases Department, National Institute for Infectious Diseases, Lazzaro Spallanzani IRCCS, Rome, Italy; Department of Experimental Medicine, University of Rome Tor Vergata, Rome, Italy; Laboratory of Virology, National Institute for Infectious Diseases, Lazzaro Spallanzani IRCCS, Rome, Italy; Department of Experimental Medicine, University of Rome Tor Vergata, Rome, Italy

## Abstract

**Background:**

Transmitted drug resistance (TDR) is still a critical aspect for the management of individuals living with HIV-1. Thus, its evaluation is crucial to optimize HIV care.

**Methods:**

Overall, 2386 HIV-1 protease/reverse transcriptase and 1831 integrase sequences from drug-naïve individuals diagnosed in north and central Italy between 2015 and 2021 were analysed. TDR was evaluated over time. Phylogeny was generated by maximum likelihood. Factors associated with TDR were evaluated by logistic regression.

**Results:**

Individuals were mainly male (79.1%) and Italian (56.2%), with a median (IQR) age of 38 (30–48). Non-B infected individuals accounted for 44.6% (*N* = 1065) of the overall population and increased over time (2015–2021, from 42.1% to 51.0%, *P* = 0.002). TDR prevalence to any class was 8.0% (B subtype 9.5% versus non-B subtypes 6.1%, *P* = 0.002) and remained almost constant over time. Overall, 300 transmission clusters (TCs) involving 1155 (48.4%) individuals were identified, with a similar proportion in B and non-infected individuals (49.7% versus 46.8%, *P* = 0.148). A similar prevalence of TDR among individuals in TCs and those out of TCs was found (8.2% versus 7.8%, *P* = 0.707).

By multivariable analysis, subtypes A, F, and CFR02_AG were negatively associated with TDR. No other factors, including being part of TCs, were significantly associated with TDR.

**Conclusions:**

Between 2015 and 2021, TDR prevalence in Italy was 8% and remained almost stable over time. Resistant strains were found circulating regardless of being in TCs, but less likely in non-B subtypes. These results highlight the importance of a continuous surveillance of newly diagnosed individuals for evidence of TDR to inform clinical practice.

## Background

HIV-1 transmitted drug resistance (TDR) is still a clinical and public health issue today because it can compromise the response to antiretroviral therapy (ART) at the individual and population level.^1–3^ As a result, testing for TDR in reverse transcriptase and protease in newly diagnosed people with HIV (PHW) is recommended by European and American guidelines as a part of the initial clinical assessment.^4,5^

The estimates of TDR rates vary substantially over time and by country.^6–9^ The TDR rate between 2014 and 2019 was reported to be stable at around 8%–10% in Europe^6^ and at around 14%–18% in the USA.^7,8^ TDR has been most detected in nucleoside reverse transcriptase inhibitors (NRTIs) and non-NRTIs (NNRTIs), while a lower prevalence is usually reported for TDR in protease inhibitors (PIs).^2,6,7,9^ So far, TDR is still rare for integrase strand transfer inhibitors (INSTI),^2,8,10^ and therefore integrase genotyping before ART initiation is not recommended unless there is suspicion of transmitted INSTI resistance or if there is a history of pre-exposure prophylaxis with cabotegravir.^5^ However, there has been an increased use of INSTIs thus, surveillance programmes to monitor TDR to this drug class are needed.

HIV subtype is another virologic factor that should be taken into consideration when a newly diagnosed individual enters into care.^4,5^ In this regard, constant monitoring of the circulation of HIV subtypes worldwide is required due to the challenges they present to diagnosis, phylogenetic reconstruction, treatment and vaccine development. The global geographical subtype distribution of HIV-1 is evolving over time and there has been a notable increase in newly emerging circulating recombinant forms (CRFs).^11,12^ In several western European countries (including Italy) in which an increase in non-B subtypes and CRFs has been reported, different frequencies of TDR have been observed over time according to subtypes and risk factors.^13–20^

In this scenario, several studies have highlighted the important role of transmission clusters (TCs) in TDR spread and subtype circulation.^13,20–24^ To date, the phylogenetic analysis represents one of the most important tools to better describe and monitor local HIV-1 epidemics, by correlating the genetic relationship of the viruses with information on demographics, transmission mode, new infections and drug resistance.^25,26^

For these considerations, this study aimed to evaluate TDR in protease, reverse transcriptase and integrase among newly diagnosed individuals followed in several clinical centres in north/central Italy from 2015 to 2021, according to subtypes and TCs.

## Methods

### Study population

Between 2015 and 2021, plasma samples from 2386 adult newly diagnosed PHW, naïve to ART, attending different counselling and testing centres in the Italian regions of Lazio and Emilia-Romagna, were tested for antiretroviral drug resistance genotyping according to routine clinical practice. All clinical and virological information used in this study was collected within 8 weeks after the initial HIV-1 diagnosis (range of weeks after HIV-1 diagnosis, 0–8).

### Ethics

This study was approved by the ethics committee of Tor Vergata Hospital (Ethics Approval No. 238/16, 14 December 2016) and L. Spallanzani National Institute for Infectious Diseases, IRCCS (Ethics Approval No. 38, 30 October 2003; Ethics Approval No. 80, 13 July 2016). The research was conducted on anonymous samples in accordance with the principles of the Declaration of Helsinki and the Italian Ministry of Health. All information, including virological and clinical data, was recorded in an anonymized database.

### HIV-1 genotyping and subtyping

For all individuals HIV-1 pol (containing the full-length protease, the first 335 reverse transcriptase codons and, if available, the full-length integrase) sequences were available at the time of diagnosis [median time (IQR) from diagnosis, 9 (2–29) days]. HIV-1 pol genotyping was performed on plasma samples through Sanger technology, as previously described.^27,28^ All samples were processed immediately on arrival in clinical laboratories. Subtypes were determined through phylogenetic analyses as previously described.^29^

### Evaluation of TDR and genotypic susceptibility score

Resistance was evaluated as TDR and genotypic susceptibility score (GSS) through HIVdb algorithm version 9.5.0 (https://hivdb.stanford.edu/). TDR was evaluated overall and over time by considering the surveillance list of mutations used in the Stanford database.^30–32^ HIV-1 strains were defined as resistant if carrying at least one TDR mutation.

GSS was evaluated for all the drugs used in clinical practice and all the first-line regimens recommended by guidelines.^4,5^ In particular, the proportion of individuals harbouring a fully susceptible strain for each drug and each regimen combination was evaluated. We followed Stanford HIV DB recommendations to re-categorize Stanford resistance interpretation into two-level categorization (susceptible versus resistant, https://hivdb.stanford.edu/page/release-notes/#hivalg). Specifically, a strain was considered susceptible when the algorithm score was equal to or less than two.

### Transmission cluster analysis

TCs were first deduced by the NJ method using all the 2386 pol sequences obtained by routine clinical practice in the period 2015–21. Only clusters with a bootstrap value >90% and an average genetic distance <0.015 were selected. The robustness of the TCs was further tested using the maximum likelihood (ML) method. The ML tree was inferred with the general time-reversible nucleotide substitution model (GTR) with gamma distribution among site rate heterogeneity, a proportion of invariable sites (GTR + I+Γ_5_),^33^ and 1000 bootstrap replicates by using MEGA 6 software.^34^ The GTR + I + Γ model was considered the best one by the MEGA 6 model test, as it showed the lowest Bayesian information criterion score. TCs were divided into small TCs (2–3 sequences, STCs), medium TCs (4–9 sequences, MTCs) and large TCs (≥10 sequences, LTCs).

### Statistical analysis

A descriptive analysis was performed on the overall population and by stratifying for HIV-1 B and non-B subtype groups. Results were presented by frequency (%) or median (first and third quartiles) for categorical and continuous variables, respectively. Comparisons between HIV-1 B and non-B subtype groups were conducted using the Mann–Whitney test for continuous variables and the chi-squared or Fisher’s exact test for categorical variables, as appropriate. Potential differences in the prevalence of TDR and HIV-1 subtypes (B versus non-B) between 2015 and 2021 were evaluated by the chi-squared test for trend. Factors associated with TDR were evaluated by uni-multivariable logistic regression analysis, using as confounders gender, age, subtype, risk factor, nationality, year of diagnosis and sequencing, viraemia and CD4 count at sequencing, state of infection (recent or not) and to be part of a TC. Regarding genotypic susceptibility per each drug and first-line regimen, to compare the proportion of individuals with susceptible GSS according to subtype (B versus non-B), chi-squared or Fisher’s exact tests were used as appropriate. The Benjamini–Hochberg method was used to correct for multiple testing at a false discovery rate of 0.05. For all statistical tests, the level of significance for the evaluation of two-sided *P* values was set at ≤0.05. All the analyses were performed using the SPSS (v.23) for Windows (SPSS Inc., Chicago, IL, USA) software.

### Sequence data

HIV-1 pol sequences from this study have been submitted to GenBank and may be accessed by the accession numbers listed in Table S1 (available as Supplementary data at *JAC* Online).

## Results

### Individuals’ characteristics

**Figure 1.**
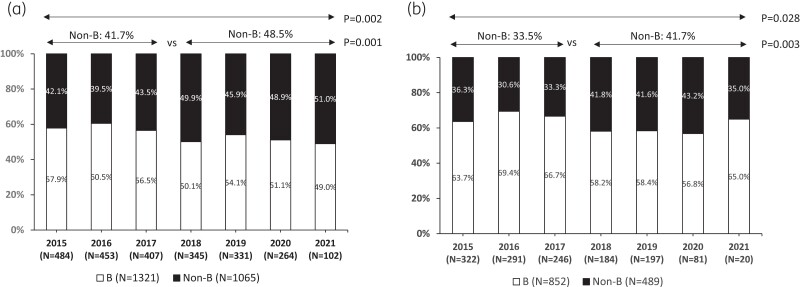
Evaluation of HIV-1 B and non-B subtype prevalence in newly diagnosed individuals over time in the overall population (a) and in Italians (b). Chi-squared test for the trend was used to evaluate potential differences in the prevalence of B and non-B subtypes over the years 2015–2021. *P*values in the figure are referred to the evaluation of potential differences during the periods 2015–2017 and 2018–2021.

A total of 2386 PHW newly diagnosed from 2015 to 2021 were included (Table 1). Most of these individuals (1887, 79.1%) were male, Italian (1341, 56.2%) and men who have sex with other men (MSM; 826, 34.6%). Recent infections accounted for 20.5% of the individuals for whom state of infection was available. More than half (*N* = 1321, 55.4%) of the individuals were infected with a B subtype, followed by CRF02_AG (195, 8.2%), F1 (148, 6.2%), A [139, 5.8%, classified as follows: A1 (126, 5.3%), A3 (5, 0.2%), A6 (8, 0.3%)], and C (139, 5.8%). The remaining individuals (444, 18.6%) were infected with other pure subtypes or CRFs. An increase in the proportion of newly diagnosed PHW with non-B subtype was found over the period 2015–2021 (from 42.1% in 2015% to 51.0% in 2021, *P* = 0.002) (Figure 1a). Among Italian individuals the proportion of non-B subtypes increased after 2017 (from 33.5% in the period 2015–2017 to 41.7% during 2018–2021, *P* = 0.003) (Figure 1b).

**Table 1.** Individuals’ characteristics

Characteristics	Overall (*N* = 2386)	B (*N* = 1321)	Non-B (*N* = 1065)	*P* value^a^
Gender, *n* (%)				<0.001
Male	1887 (79.1)	1121 (84.9)	766 (71.9)	
Female	443 (15.6)	125 (12.0)	280 (26.7)	
Unknown	56 (5.3)	75 (3.1)	19 (1.4)	
Geographic area, *n* (%)				<0.001
Italy	1341 (56.2)	852 (64.5)	489 (45.9)	
Africa	187 (7.8)	19 (1.4)	168 (15.8)	
Europe	173 (7.3)	109 (8.3)	64 (6.0)	
America	43 (1.8)	21 (1.6)	22 (2.1)	
Asia/Oceania	151 (6.3)	60 (4.5)	91 (8.5)	
Unknown	491 (20.6)	260 (19.7)	231 (21.7)	
Risk factor, *n* (%)				<0.001
MSM	826 (34.6)	556 (42.1)	270 (25.4)	
Heterosexual	573 (24.0)	254 (19.2)	319 (30.0)	
IDU	82 (3.4)	55 (4.2)	27 (2.5)	
Sexual	193 (8.1)	90 (6.8)	103 (9.7)	
Other	10 (0.4)	5 (0.4)	5 (0.5)	
Unknown	702 (29.4)	361 (27.3)	341 (32.0)	
Age (years), median (IQR)	38 (30–48)	39 (31–49)	37 (29–45)	<0.001
CD4 cell count (cells/mm^3^), median (IQR) (*N* = 2337)	297 (115–500)	294 (101–501)	304 (125–500)	0.554
Viral load (log_10_ copies/mL), median (IQR) (*N* = 2263)	5.0 (4.4–5.6)	4.9 (4.4–5.5)	5.1 (4.5–5.7)	<0.001
Year of diagnosis, median (IQR) (*N* = 2325)	2017 (2016–2019)	2017 (2015–2019)	2017 (2016–2019)	<0.001
Year of GRT, median (IQR)	2017 (2016–2019)	2017 (2016–2019)	2017 (2016–2019)	0.003
State of infection, *n* (%)^b^				0.303
Chronic	817 (34.2)	462 (35)	355 (33.3)	
Recent	488 (20.5)	279 (21.1)	209 (19.6)	
Unknown	1081 (45.3)	580 (43.9)	501 (47.0)	
TDR, *n* (%)				
Overall	191 (8.0)	126 (9.5)	65 (6.1)	0.002
PI	30 (1.3)	22 (1.7)	8 (0.8)	0.046
NRTI	62 (2.6)	46 (3.5)	16 (1.5)	0.003
NNRTI	114 (4.8)	72 (5.5)	42 (3.9)	0.086
INSTI^c^	6 (0.3)	4 (0.2)	2 (0.5)	0.415
Involvement in TC, *n* (%)				
In cluster	1155 (48.4)	657 (49.7)	498 (46.8)	0.148
Out of cluster	1231 (51.6)	664 (50.3)	567 (53.2)	
Type of TC, *n* (%)^d^				<0.001
Small TC (2–3 sequences)	448 (18.8)	294 (22.3)	154 (14.5)	
Medium TC (4–9 sequences)	430 (18.0)	264 (20.0)	166 (15.6)	
Large TC (≥10 sequences)	277 (11.6)	99 (7.5)	178 (16.7)	

GRT, genotypic resistance test; IDU, injection drug user; INSTI, integrase strand transfer inhibitor; MSM, men who have sex with other men; NNRTI, non-nucleoside reverse transcriptase inhibitor; NRTI, nucleos(t)ide reverse transcriptase inhibitor; PI, protease inhibitor; TCs, transmission clusters; TDR, transmitted drug resistance.

^a^By *χ*^2^ test or Fisher’s exact test, as appropriate (qualitative variables), and Wilcoxon–Mann–Whitney test (quantitative variables).

^b^Individuals were defined as recently infected by: (i) clinical/laboratory signs of primary HIV infection (HIV-1 RNA levels >10 000 copies/mL and negative or indeterminate HIV-1 antibody test); (ii) a documented negative HIV-1 test performed within 6 months before the HIV-1 diagnosis; and (iii) an antibody avidity index ≤0.80 (test performed only in clinically AIDS free individuals).^17^

^c^Analysis performed on the 1831 integrase sequences available.

^d^Analysis performed by considering the 1155 individuals involved in the TCs.

An increase over time of newly diagnosed PHW with non-B subtype was found overall and within the Italian population (Figure 1). In particular, in the overall population the proportion of non-B subtypes increased from 41.7% during 2015–2017 to 48.5% between 2018 and 2021 (*P* = 0.001, Figure 1a). In the same way, among Italian individuals the proportion of non-B subtypes increased from 33.5% to 41.7% (*P* = 0.003) (Figure 1b).

Regarding risk factors, while individuals infected with B subtype were predominantly MSM (556, 42.1%), non-B subtype-infected individuals mainly reported heterosexual contact (319, 30.0%) (Table 1).

### Prevalence and temporal trend of transmitted drug resistance

Overall, 191 (8.0%) individuals carried a TDR virus in the period 2015–2021; most of them showed a single resistance mutation (*N* = 159, 83.2%). TDR was higher in B subtype-infected individuals than in those infected with non-B subtypes (9.5% versus 6.1%, *P* = 0.002; Table 1).

**Figure 2.**
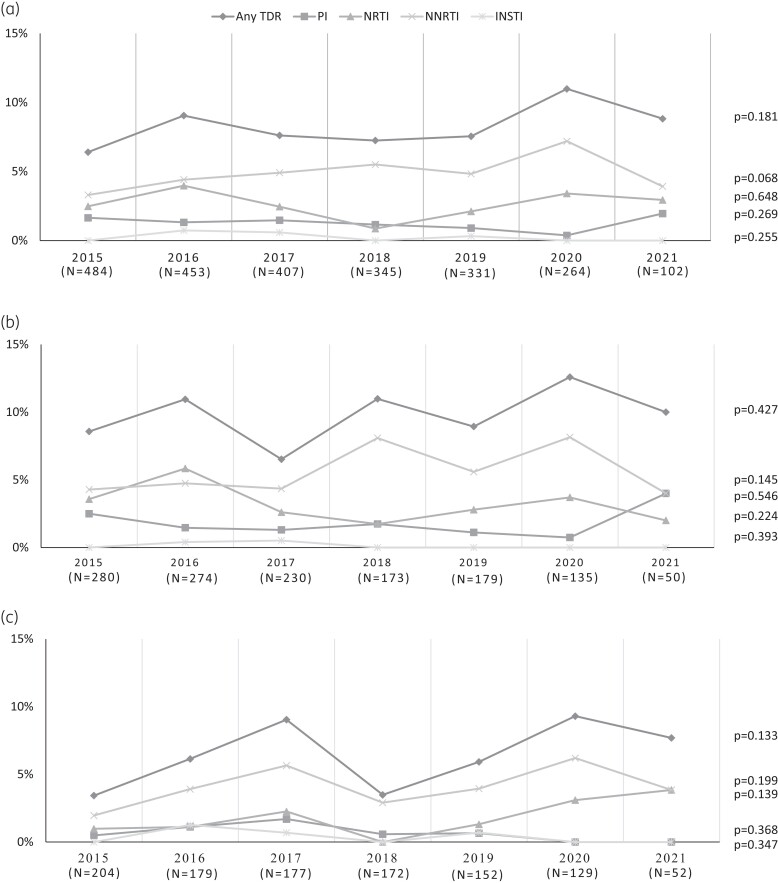
Prevalence of TDR in Italian PHW, diagnosed in the period 2015–2021, according to calendar year and drug classes. (a) Prevalence of TDR over time in the overall population. (b) Prevalence of TDR over time in individuals infected with the HIV-1 B subtype. (c) Prevalence of TDR over time in individuals infected with HIV-1 non-B subtypes. TDR was evaluated by considering the surveillance list of mutations used in HIVdb.^31–33^ The *P* values were calculated using the chi-squared test for trend.

Analysing the TDR temporal trend, no significant changes in the prevalence of TDR to any class were found between 2015 and 2021 (2015–2021: 6.4%–8.8%, *P* = 0.181) (Figure 2a). The same situation was found when the specific drug classes were considered (Figure 2a). Similarly, no significant changes in TDR prevalence were found by stratifying for HIV-1 subtype (B subtype, 2015–2021, 8.6%–10.0%, *P* = 0.427; non-B subtypes, 2015–2021, 3.4%–7.7%, *P* = 0.133; Figure 2b and c).

**Figure 3.**
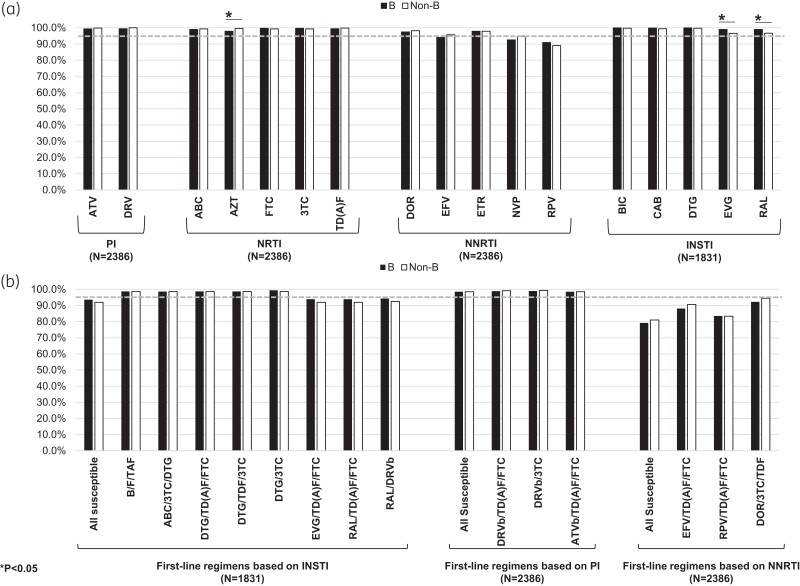
Genotypic susceptibility to antiretrovirals among HIV-1 B and non-B subtype in newly diagnosed individuals. (a) Proportion of individuals harbouring fully susceptible virus per each ARV. (b) Proportion of individuals harbouring fully susceptible virus per each first-line regimen used in clinical practice.^4,5^ The dotted line indicates a proportion of 95%.

GSS was also estimated per each drug and first-line regimen used in clinical practice (Figure 3). Overall, most ARVs showed a genotypic full activity in at least 95% of individuals; nevirapine and rilpivirine were the only exceptions showing a proportion of full activity in 93.6% and in 90.1% of individuals, respectively. Similarly, first-line regimens (except for regimens based on efevirenz or rilpivirine) showed a genotypic full activity in at least 95% of individuals regardless of subtype (Figure 3a and b). A difference in the proportion of susceptibility according to subtype was found for the first-generation INSTIs with a lower susceptibility in non-B infected individuals compared to B infected (B versus non-B: EVG, 99.0% versus 96.5, *P* = 0.017; RAL: 99.0% versus 96.7%, *P* = 0.017, Figure 3a).

### Transmission clusters and their role in TDR

Overall, we identified 300 TCs, involving 1155 of the 2386 newly diagnosed individuals analysed (48.4%), with a similar proportion in newly diagnosed individuals with B subtype and those with non-B subtypes (49.7% versus 46.8%, *P* = 0.148). Most of the individuals were native (65.5%), with a median (IQR) age of 38 (30–47), and MSM (38.7%). Their median (IQR) CD4 cell count was 325 (129–532) cells/mm^3^, while their viraemia was 5.1 (4.5–5.7) log_10_ copies/mL. The characteristics of these 1155 individuals involved in TCs were similar regardless of subtype (data not shown).

Looking at resistance, among the 300 TCs found, 43 (including 240 individuals) involved at least one subject with TDR (95 individuals, corresponding to 49.7% of the entire TDR population). Specifically, 18 TCs (41.9%) included only one patient with TDR, 18 (41.9%) were entirely composed of individuals with TDR, while the remaining seven (16.3%) included more than one individual with TDR.

By evaluating the characteristics of the 240 individuals involved in these 43 TCs according to HIV-1 subtype, compared to individuals infected with non-B subtypes, those infected with B subtype were mostly men, had a higher CD4 cell count and a lower viral load, were diagnosed in more recent years, were involved mainly in small and medium TCs, and were more likely infected with viruses harbouring TDR (Table S2).

### Prevalence of TDR mutations in the overall population and in the TCs

**Figure 4.**
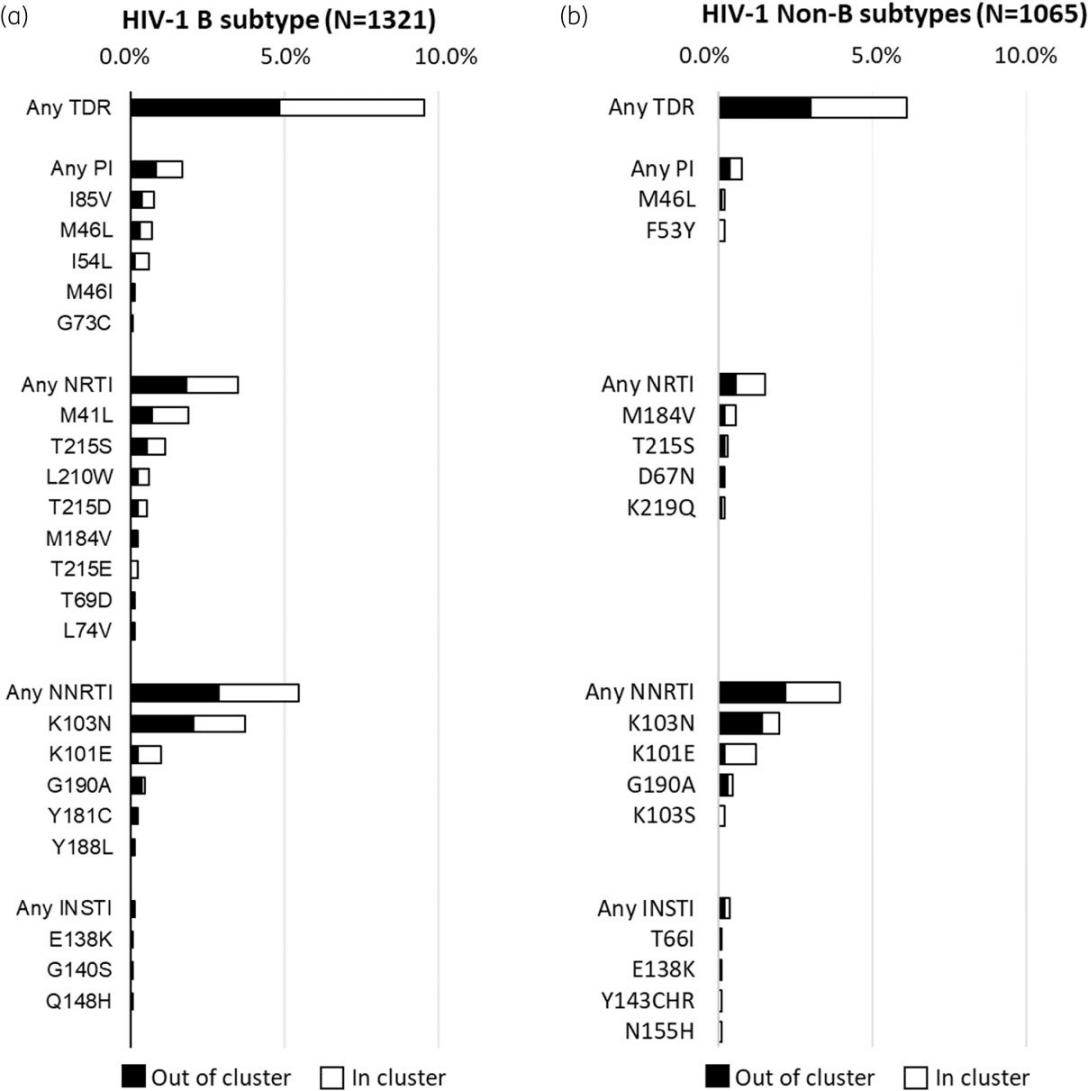
Prevalence of drug resistance mutations in HIV-1 B (a) and non-B subtypes (b) according to drug classes and presence or not in TCs. Surveillance mutations are reported that used HIVdb^31–33^ present in at least two individuals for PI and RTI and in at least one individual for INSTI. Each bar is divided according to (i) the presence of drug resistance mutations in clusters (in white) and (ii) the presence of drug resistance mutations out of clusters (in black).

The prevalence of TDR mutations in the overall population and in the TCs according to drug classes and subtypes is shown in Figure 4. Analysing resistance in HIV-1 B subtype-infected individuals, 3.5% of them (46/1321) harboured mutations associated with resistance to NRTIs; of note, 1.7% of these individuals (22/1321) were involved in TCs. The NRTI mutation M41L had the highest prevalence in the overall B subtype-infected population (1.9%: individuals involved in TCs, 1.2%; individuals not included in TCs, 0.7%), followed by T215S (1.1%: 0.6% and 0.5%) and L210W (0.6%: 0.4% and 0.2%). Interestingly, in non-B subtype-infected individuals NRTI mutations accounted only for 1.5% of the overall population; 0.9% of these mutations (10/1065) were found in TCs. M184V accounted for 0.2% out of TCs in B subtype and 0.6% in non-B subtypes (in TCs, 0.4%; out of TCs, 0.2%).

Concerning NNRTI resistance, 5.4% (72/1321) and 3.9% (42/1065) of the B and non-B subtype-infected individuals carried at least one mutation to this class, respectively. Of note, 2.6% of B (34/1321) and 1.8% of non-B (19/1065) subtype-infected individuals were involved in TCs. In both B and non-B subtype groups, the most prevalent NNRTI mutations (either in individuals included or in those non-included in TCs) were K103N (B subtype, 3.7%; non-B subtype, 2.0%) and K101E (1.0%; 1.2%), followed by G190A (0.5% both). Of note, the mutations V106I and E138A, not reported in the surveillance list but considered in the GSS estimation, were found with a considerable frequency (3.5% and 7.0%, respectively; Table S3). In the overall B and non-B subtype groups, mutations associated with resistance to PIs were observed with a prevalence of 1.6% (0.8% in both individuals involved in TCs and not included in TCs) and 0.8% (0.4% in both TCs and out of TCs); I85V was the most prevalent PI mutation found only in the B group (0.8%: 0.4% in both TCs and out of TCs), followed by M46L (0.7%; 0.4% and 0.3%) This mutation was also found in non-B subtypes (0.2%; 0.1% in both TCs and out of TCs).

TDR to INSTIs was rare [overall, *n* = 6 (0.3%); within B subtype, *n* = 2 (0.2%); within non-B subtype, *n* = 4 (0.4%)]. Regarding the B subtype, no TDR to this drug class was found in TCs, while in non-B subtypes, 0.2% of TDR was found in both TCs and out of TCs. Among these six individuals harbouring INSTI resistance, one had resistance to NRTIs, one had resistance to NNRTIs and another one had resistance to both NRTIs and NNRTIs (Table S4). Two individuals were part of TCs.

### Factors associated with TDR

Univariate and multivariate logistic regression models were performed to identify potential predictors of TDR (Table 2). The results showed that in our population, being part of TCs was not a predictor of TDR [odds ratio, AOR (95% CI): 1.1 (0.8–1.4); *P* = 0.701], whereas there was a negative association between the presence of TDR and individuals infected with a CRF02_AG recombinant form [AOR (95% CI): 0.3 (0.2–0.8); *P* = 0.008], F [AOR (95% CI): 0.3 (0.1–0.7); *P* = 0.011] and A [AOR (95% CI): 0.4 (0.1–0.9); *P* = 0.032] subtypes compared with those infected with a B subtype (Table 2).

**Table 2.** Factors associated with the detection of TDR in c-ART naïve individuals

Variables	Odds ratio of detecting TDR			
	Crude		Adjusted^a^	
	OR (95% CI)	*P* value	OR (95% CI)	*P* value
Gender				
Male^b^	1			
Female	1.1 (0.8–1.7)	0.515		
Unknown	0.7 (0.2–2.1)	0.538		
Age	1.0 (1.0–1.0)	0.903		
Subtype	1			
B^b^				
CRF02_AG	0.4 (0.2–0.8)	0.009	0.3 (0.2–0.8)	0.008
F1	0.3 (0.1–0.7)	0.010	0.3 (0.1–0.7)	0.011
C	0.7 (0.4–1.4)	0.368	0.7 (0.4–1.4)	0.326
A	0.4 (0.1–0.9)	0.025	0.4 (0.1–0.9)	0.032
Other	0.9 (0.6–1.3)	0.637	0.9 (0.6–1.4)	0.768
Risk factor				
MSM^b^	1		1	
Heterosexual	0.7 (0.5–1.0)	0.069	0.8 (0.5–1.2)	0.321
IDU	0.8 (0.3–1.9)	0.618	0.9 (0.4–2.1)	0.798
Sexual	1.0 (0.6–1.8)	0.873	1.1 (0.7–2.0)	0.623
Other	1.1 (0.1–9.0)	0.909	1.5 (0.2–12.5)	0.705
Unknown	0.9 (0.6–1.3)	0.493	1.0 (0.7–1.4)	0.966
Nationality				
Italian^b^	1			
non-Italian	1.0 (0.7–1.4)	0.831		
Unknown	1.0 (0.7–1.5)	0.948		
Year at genotyping	1.1 (1–1.1)	0.181		
State of infection				
Chronic^b^	1			
Recent	1.0 (0.6–1.4)	0.857		
Unknown	0.8 (0.6–1.2)	0.270		
Viraemia at genotyping				
<100 000^b^	1			
100 000–1 000 000	0.8 (0.6–1.2)	0.304		
>1 000 000	0.9 (0.6–1.5)	0.662		
unknown	1.5 (0.9–2.7)	0.154		
CD4 at genotyping				
<200^b^	1		1	
201–350	0.7 (0.4–1.1)	0.110	0.7 (0.4–1.1)	0.110
350–500	1.0 (0.7–1.6)	0.918	1.0 (0.7–1.6)	0.913
>500	1.4 (1–2)	0.080	1.4 (0.9–2.0)	0.102
Unknown	0.2 (0.0–1.8)	0.164	0.2 (0.0–1.6)	0.138
TCs				
no TC versus TC>=2	1.1 (0.8–1.4)	0.701		

^a^Only variables significant at univariable analysis (*P* < 0.1) were retained in multivariable models.

^b^Reference (dummy).

## Discussion

To gain further insight into the time trends of subtype distribution, TDR and TCs, we have described the epidemiological and molecular characteristics of 2386 newly diagnosed individuals with HIV-1 attending several counselling and testing centres in north and central Italy between 2015 and 2021. The present study represents an update of a previous analysis on protease and reverse transcriptase,^17^ implemented with the evaluation of resistance to INSTIs in a considerable number of integrase sequences (more than 1800). We found an increase of non-B subtypes over time, confirming the increasing trend observed between 2000 and 2014,^17^ up to the point that, today, new diagnoses with non-B subtypes represent about 50% of the cases, as already observed in another Italian Cohort.^16^ The increase was confirmed not only in the overall population but also among Italian subjects, around 40% of whom were infected with non-B subtypes viral strains in recent years. The most common non-B subtypes were CRF02_AG, F1, A and C, similar to those generally observed in central Europe.^24^ However, it is of note that subtype A, specifically A6, one of the factors associated with the failure to the new treatment strategy based on long-acting combination with cabotegravir and rilpivirine, was found only in eight (0.3%) individuals among the overall population, while most of individuals were infected with subtype A1 (5.3% of the overall population), which does not seem be associated with failure to the combination.^35–37^

These findings highlight the importance of providing accurate information about the resistance and the HIV-1 subtype.

With regards to resistance, the overall TDR prevalence in Italy between 2015 and 2021 was 8%, similar to what was previously observed by our group^17^ and by other studies focused on TDR prevalence in Italy from 2013 to 2018^6,16^ or, more generally, in Europe between 2014 and 2019.^7^ TDR prevalence remained almost stable over time, overall and in both B and non-B subtypes; this was in contrast to the trend observed in our previous study in which TDR prevalence increased in non-B subtypes and decreased in B subtypes. On the other hand, a constant trend of TDR prevalence (although higher than found in our study) was recently observed in Europe and the USA.^6,8^ The stable trend over time can be explained by the fact that TDR resistance was mainly due to the presence of NNRTI mutations (particularly the K103N) in both HIV-1 B and non-B subtypes despite the change of landscape of ART prescription from efavirenz-based regimens to INSTI-based regimens.^4,5^ Unlike NRTI resistance mutations (such as, for example, M184V that in our cohort had a very low prevalence), the mutations associated with NNRTI resistance might only have a minimal impact on viral fitness and have longer intra-host persistence.^38^

By analysing TDR to INSTIs, we found a very low prevalence (<0.5%), thus confirming what has been observed so far in other studies.^2,8,10^ The integrase resistance mutations transmitted were mainly related to the first-generation INSTIs raltegravir and elvitegravir. Among the six individuals with TDR to INSTIs, three of them harboured resistance also to NRTIs and/or NNRTIs. It is noteworthy that one of these six individuals harboured the INSTI pattern G140S + Q148H that confers intermediate-/high-level resistance to all the INSTIs.^39,40^ These findings highlight the importance of performing a GRT in newly diagnosed individuals not only in protease and reverse transcriptase, but also in integrase in the light of long-term successful management of these individuals.

By analysing genotypic susceptibility to antiretrovirals, we found a lower prevalence of susceptibility (≤95%) for first-line regimens based on NNRTIs, explained by the presence of the mutation K103N for efavirenz, the polymorphic mutations at the position E138 for rilpivirine and the mutation V106I for doravirine (Figure 4 and Table S3). Regarding the mutations at positions E138 and V106I, these are not considered in the surveillance list^30^ but they are considered in the evaluation of genotypic susceptibility. In fact, mutations at position E138 play a role in the rilpivirine resistance and (especially the polymorphic mutation E138A, present in our population study at around 7%; Table S3) in virological failure^41,42^ while the accessory mutation V106I can be associated with doravirine resistance.^43^ We also found a lower prevalence of susceptibility to the first-generation INSTIs, while a high genotypic susceptibility was found for the second-generation INSTIs as described before.

This study also evaluated the contribution of TCs in the spread of HIV-1 and TDR. Overall, 300 TCs were identified, involving about half the individuals of the population analysed, with a similar proportion of B and non-B subtype infections. The characteristics of these 1155 individuals involved in TCs were similar regardless of subtypes (data not shown). Most of them were native, MSM and with a median age of 38, as confirmed by our previous^17^ and other European studies.^6,13^

Regarding TDR in TCs, mutations associated with resistance to NRTIs, in particular those associated with thymidine analogues, were predominant in B subtype TCs, confirming the frequent transmission of viruses containing these mutations in Europe.^44,45^ By contrast, thymidine analogues were rarely found in non-B TCs. A similar scenario was found for the mutations associated with resistance to PIs. Mutations associated with NNRTIs, such as K103N/S and K101E were present in both B and non-B subtypes, confirming, at least in the case of K103N, a more frequent transmission clustering.^46^

We finally evaluated factors related to the presence of TDR. Similar to our previous study,^17^ as the multivariate logistic regression model did not identify factors associated with TDR, with the exception of a negative role of some subtypes (such as CRF02_AG, F1 and A), we were unable to identify positive predictors of drug resistance.

As with any other observational study, our data may have some limitations. First, our study population might not be representative of the overall Italian population because it is strictly related to the Italian regions of Lazio and Emilia-Romagna. However, the high prevalence of non-B subtypes found in our population in recent years is in line with other Italian^16,47,48^ and European^10^ studies, suggesting a good reproducibility of our results with respect to large European contexts. Second, genotyping was performed through Sanger technology. This test has fairly limited sensitivity but it was the technique used in clinical routine during the time period described. Finally, the number of sequences in the last 2 years of observation was lower; this could be related to the effect of the COVID pandemic.

In conclusion, the present study shows that, in recent years, TDR to PIs and RTIs has remained constant at about 8% in Italy, a fact that is mainly due to NNRTI mutations. Some cases of TDR to INSTIs have also been detected. Resistant strains were found circulating regardless of being in TCs, but these were less likely in non-B subtypes. Thus, our findings reinforce the importance of evaluating HIV-1 resistance in newly diagnosed individuals not only in protease and reverse transcriptase but also in integrase as a part of routine testing in clinical practice and national surveillance programmes. These programmes are needed to continuously monitor the presence of TDR, as well as for providing the HIV molecular information (such as HIV subtypes and TCs) that is needed for a correct knowledge of national HIV molecular epidemiology.

## Supplementary Material

dkae189_Supplementary_Data

## Italian HIV Drug Resistance Group

University of Rome ‘Tor Vergata’, Rome, Italy: F. Ceccherini-Silberstein, M.C. Bellocchi, L. Carioti, M.M. Santoro, V. Svicher; Policlinic of Rome Tor Vergata, Rome: M. Andreoni, M. Iannetta, A. Bertoli, L. Sarmati, V. Malagnino; E. Teti; Saint Camillus International University of Health Sciences, Rome, Italy: D. Armenia; National Institute for Infectious Diseases ‘L. Spallanzani’, IRCCS, Rome, Italy: A. Antinori, F. Baldini, R. Bellagamba, G. Berno, M. Camici, S. Cicalini, F. De Zottis, R. Esvan, L. Fabeni, F. Forbici, M. Fusto, R. Gagliardini, S. Gebremeskel, F. Gili; E. Girardi, E. Grilli, S. Grisetti, I. Mastrorosa, V. Mazzotta, A. Mondi, N. Orchi; S. Ottou, C. Pinnetti, S. Pittalis; D. Pizzi, M. Plazzi, A. Vergori; San Gallicano Hospital, IRCCS, Rome, Italy: A.R. Buonomini, M. Giuliani, A. Latini, A. Pacifici; Bambino Gesù Children's Hospital, Rome, Italy: C.F. Perno; La Sapienza University, Rome, Italy: V. Belvisi, C. Del Borgo, A. Carraro, M. Lichtner, R. Marocco; University Hospital, University of Modena and Reggio Emilia, Modena, Italy: V. Borghi, C. Mussini, W. Gennari.

This paper was in part presented as an oral presentation at the European Meeting on HIV & Hepatitis, 7–9 June 2023, Rome, Italy (Abstract 72).

## Supplementary data

Tables S1 to S4 are available as Supplementary data at *JAC* Online.
