# Supplementary material for: HIV-1 transmitted drug resistance in newly diagnosed individuals in Italy over the period 2015–21
Source: J Antimicrob Chemother. 2024 Jul 19;79(9):2152–62. doi: 10.1093/jac/dkae189 (PMC11368429; doi:10.1093/jac/dkae189)
Supplement: dkae189_Supplementary_Data [file dkae189_supplementary_data.docx]

**Supplementary table 1.** GenBank accession numbers of HIV-1 pol sequences analyzed.

| Pol Region | Accession Number |
| --- | --- |
| Protease & reverse transcriptase | OR993476, OR993002, OR993641, OR993399, OR993466, OR993470, OR993468, OR993472, OR993462, OR993522, OR993649, OR993645, OR993647, PP451183 - PP453555 |
| Integrase | OR993388, OR993419, OR993009, OR993011, OR993604, OR993583, OR993093, OR993057, OR993493, OR993598, OR993370, OR993587, OR993163, OR993296, OR993175, OR993300, OR992971, OR993509, OR993505, OR993628, OR993203, OR993441, OR993091, OR993392, OR993346, OR993105, OR993227, OR993083, OR993037, OR993065, OR993061, OR993007, OR993017, OR993071, OR993253, PP449387 - PP451182 |

**Supplementary Table 2:** Characteristics of HIV-1 infected individuals involved in the 43 TDR TCs

| **Characteristics** | **Overall TCs**  **(N=43)** | **B subtype TCs**  **(N=27)** | **Non-B TCs**  **(N=16)** | **p-value^a^** |
| --- | --- | --- | --- | --- |
| **Subjects involved in TCs, n** | 240 | 126 | 114 |  |
| **Gender, n (%):** |  |  |  |  |
| Male | 208 (86.7) | 113 (89.7) | 95 (83.3) | **0.031** |
| Female | 26 (10.8) | 13 (10.3) | 13 (11.4) |  |
| Unknown | 6 (2.5) | 0 (0.0) | 6 (5.3) |  |
| **Age (years), median (IQR)** | 38 (29-48) | 37 (29-45) | 40 (29-51) | 0.152 |
| **CD4 cell count (cells/mm^3^), median (IQR)** | 314 (135-529) | 378 (180-547) | 286 (115-473) | **0.046** |
| **Viral load (log_10_ copies/mL), median (IQR)** | 5.2 (4.5-5.7) | 5.0 (4.4-5.5) | 5.4 (4.7-6.0) | **<0.001** |
| **Year of diagnosis, median (IQR)** | 2017 (2016-2019) | 2017 (2016-2019) | 2018 (2016-2019) | **0.022** |
| **Year of GRT, median (IQR)** | 2017 (2016-2019) | 2017 (2016-2019) | 2018 (2016-2019) | **0.03** |
| **TDR, n (%):** |  |  |  |  |
| Any drug class | 95 (39.6) | 62 (49.2) | 33 (28.9) | **<0.001** |
| PI | 15 (6.3) | 11 (8.7) | 4 (3.5) | 0.095 |
| NRTI | 32 (13.3) | 22 (17.5) | 10 (8.8) | **0.048** |
| NNRTI | 53 (22.1) | 34 (27.0) | 19 (16.7) | 0.054 |
| INSTI^b^ | 2 (1.0) | 0 (0.0) | 2 (2.4) | 0.111 |
| **Geografic area, n (%)** |  |  |  |  |
| Italy | 167 (69.6) | 92 (73.0) | 75 (65.8) | 0.169 |
| Africa | 1 (0.4) | 0 (0.0) | 1 (0.9) |  |
| Europe | 11 (4.6) | 2 (1.6) | 9 (7.9) |  |
| America | 14 (5.8) | 9 (7.1) | 6 (4.4) |  |
| Asia/Australia/Oceania | 2 (0.8) | 1 (0.8) | 1 (0.9) |  |
| Unknown | 45 (18.8) | 22 (17.5) | 23 (20.2) |  |
| **Risk factor, n (%):** |  |  |  |  |
| MSM | 116 (48.3) | 67 (53.2) | 49 (43.0) | 0.195 |
| Heterosexual | 38 (15.8) | 17 (13.5) | 21 (18.4) |  |
| IDU | 2 (0.8) | 2 (1.6) | 0 (0.0) |  |
| Sexual | 15 (6.3) | 5 (4.0) | 10 (8.8) |  |
| Perinatal | 1 (0.4) | 0 (0.0) | 1 (0.9) |  |
| Unknown | 68 (28.3) | 35 (27.8) | 33 (28.9) |  |
| **Type of TC, n (%)** |  |  |  |  |
| Small TC (2-3 sequences) | 59 (24.6) | 41 (32.5) | 18 (15.8) | **<0.001** |
| Medium TC (4-9 sequences) | 42 (17.5) | 27 (21.4) | 15 (13.2) |  |
| Large TC (≥10 sequences) | 138 (57.9) | 58 (46.0) | 81 (71.1) |  |

^a^By Chi-square test (qualitative variables) or Wilcoxon-Mann-Whitney (quantitative variables). ^b^Analysis on 192 individuals

TC, transmission cluster; IQR, interquartile range; GRT, genotypic resistance test; TDR, transmitted drug resistance; PI, protease inhibitor, NRTI, nucleos(t)ide reverse transcriptase inhibitor; NNRTI, non-nucleoside reverse transcriptase inhibitor; INSTI, integrase strand transfer inhibitor; MSM: men who have sex with men; IDU, injection drug user.

| **Additional resistance associated mutations listed for genotypic susceptibility score**  **(GSS, HIVdb algorithm ver 9.5)** | **Frequency n (%)**  **(N=2386)** |  |
| --- | --- | --- |
| **PI** |  |  |
| L10F | 4 (0.2) |  |
| K20AIMRVT | 10 (0.4) |  |
| L33F | 13 (0.5) |  |
| M46V | 1 (0.04) |  |
| G48E | 1 (0.04) |  |
| I50N | 1 (0.04) |  |
| N88T | | 1 (0.04) |
| L89V | 2 (0.1) |  |
| **NRTI** |  |  |
| A62V | 24 (1.0) |  |
| K65N | 1 (0.04) |  |
| **NNRTI** |  |  |
| A98G | 2 (0.1) |  |
| L100F | 1 (0.04) |  |
| K101QH | 3 (0.1) |  |
| V106I | 84 (3.5) |  |
| V108I | 10 (0.4) |  |
| E138AGKPQ^a^ | 203 (8.5) |  |
| V179DENIT | 105 (4.4) |  |
| Y181F | 1 (0.04) |  |
| H221Y | 5 (0.2) |  |
| F227L | 1 (0.04) |  |
| M230I | 1 (0.04) |  |
| L234I | 1 (0.04) |  |
| K238T | 5 (0.2) |  |
| Y318FI | 12 (0.5) |  |
| N348I | 1 (0.04) |  |
| **INSTI^b^** |  |  |
| T97A | 1 (0.04) |  |
| N155IKMT | 2 (0.1) |  |
| E157Q | 1 (0.04) |  |

**Supplementary table 3.** Prevalence of additional resistance associated mutations in cART naïve individuals included for calculation of genotypic susceptibility score (GSS, HIVdb ver 9.5) of the drugs currently used in Clinical practice.

^a^E138A was the most prevalent mutation at this position (n=167, 7.0%). bCalculated for 1831 individuals with available integrase GRT.

**Supplementary Table 4.** Characteristics of the six newly diagnosed individuals harbouring HIV integrase resistant strains.

| **ID** | **Subtype** | **TC** | **Viral load^c^** | **Major resistance mutations** | | | | **Drug resistance interpretation^d^** | | | | |
| --- | --- | --- | --- | --- | --- | --- | --- | --- | --- | --- | --- | --- |
|  |  |  |  | **PI** | **NRTI** | **NNRTI** | **INSTI** | **EVG** | **RAL** | **DTG** | **CAB** | **BIC** |
| 1 | A3 | Yes^a^ | 7.0 | None | None | None | Y143CHR | P | H | S | P | S |
| 2 | CRF64_BC | No | 4.5 | None | None | K103N | T66I | H | L | S | P | S |
| 3 | CRF71_BF | Yes^b^ | 4.9 | None | M184V | None | N155H | H | H | P | L | P |
| 4 | B | No | 4.9 | None | T215S | M230L | G140S+  Q148H | H | H | I | H | I |
| 5 | C | No | NA | None | None | None | E138K | L | L | P | P | P |
| 6 | B | No | NA | None | None | None | E138K | L | L | P | P | P |

^a^Five individuals in cluster; integrase sequence was available for three individuals; only one of them had Y143CHR mutation. ^b^Eigtheen individuals in cluster; 2/18 individuals had M184V mutation; integrase sequences were available for 17 individuals, only one of them had N155H & M184V. ^c^Log_10_ copies/mL; ^d^Susceptibility of each integrase inhibitor was estimated according to the genotype interpretation obtained by the HIVDb algorithm version 9.5.0 (https://hivdb.stanford.edu). The following levels of resistance have been identified: P, Potential low-level resistance; S, susceptible; L, Low-level resistance; I, Intermediate resistance; H, High level resistance.

PI, protease inhibitor, NRTI, nucleos(t)ide reverse transcriptase inhibitor; NNRTI, non-nucleoside reverse transcriptase inhibitor; INSTI, integrase strand transfer inhibitor; EVG, elvitegravir; RAL, raltegravir; DTG, dolutegravir; CAB, cabotegravir; BIC, bictegravir.
